# Ethnic Differences in Response to COVID-19: A Study of American-Asian and Non-Asian College Students

**DOI:** 10.3390/bs13040325

**Published:** 2023-04-10

**Authors:** Yijun Zhao, Yi Ding, Hayet Chekired, Ying Wu, Qian Wang

**Affiliations:** 1 Computer and Information Sciences Department, Fordham University, New York, NY 10023, USA; hchekired@fordham.edu; 2 Graduate School of Education, Fordham University, New York, NY 10023, USA; yding4@fordham.edu (Y.D.); ywu135@fordham.edu (Y.W.); 3 School of Engineering, Manhattan College, Riverdale, NY 10471, USA; qian.wang@manhattan.edu

**Keywords:** COVID-19, university student, Asian, non-Asian, coping patterns, risk factors, supervised machine learning, SHAP method

## Abstract

Asian American students have experienced additional physical and emotional hardships associated with the COVID-19 pandemic due to increased xenophobic and anti-Asian discrimination. This study investigates different coping patterns and risk factors affecting Asian and non-Asian college students in response to COVID-19 challenges by studying the differences in their responses within four domains after the onset of the pandemic: academic adjustment, emotional adjustment, social support, and discriminatory impacts related to COVID-19. We first employed a machine learning approach to identify well-adjusted and poorly adjusted students in each of the four domains for the Asian and non-Asian groups, respectively. Next, we applied the SHAP method to study the principal risk factors associated with each classification task and analyzed the differences between the two groups. We based our study on a proprietary survey dataset collected from U.S. college students during the initial peak of the pandemic. Our findings provide insights into the risk factors and their directional impact affecting Asian and non-Asian students’ well-being during the pandemic. The results could help universities establish customized strategies to support these two groups of students in this era of uncertainty. Applications for international communities are discussed.

## 1. Introduction

Since the outbreak of COVID-19, xenophobic and anti-Asian, specifically anti-Chinese, attitudes and rhetoric have increased exponentially both in the media and in daily interactions [1,2]. Because COVID-19 was first reported in Wuhan, China [3], negative coverage in the news media, as well as the public’s lack of understanding of the virus, has fueled increasing anti-Chinese and anti-Asian sentiments. For example, President Trump repeatedly labeled COVID-19 as the “Kung Flu” [4,5]. Similarly, news articles covering COVID-19 updates included photos of Asians and Chinatowns that were not directly related to the articles themselves [6].

Increasing anti-Chinese and anti-Asian sentiments have led to increased discrimination and racism toward Asian-American individuals and communities [7,8,9]. Dylan Scott [4] reported that researchers from San Francisco identified more than 1000 reported cases of xenophobia against the Chinese American community between 28 January and 24 February 2020. Because Asian Americans have been harassed and physically assaulted due to racist attitudes surrounding the COVID-19 pandemic, State Attorney General James launched a hotline for New Yorkers to report hate crimes and bias-based incidents [10]. Such xenophobic and anti-Asian attitudes and reactions in the public have posed additional physical hardships and emotional burdens to students of Asian ethnicities [11].

The overarching goal of this study is to compare and contrast similarities and differences in coping patterns and risk factors affecting Asian and non-Asian college students in response to COVID-19 challenges in four domains after the onset of the pandemic: academic adjustment, emotional adjustment, social support, and discriminatory impacts. The dependent variables employed in this study are well-established psychological metrics, including the Ways of Coping Questionnaire (WAYS [12]), the Student Adaptation to College Questionnaire (SACQ [13]), and the Perceived Stress Scale (PSS [14]). We first administered an extensive survey to 517 U.S. college students during the initial peak of the pandemic, with questions measuring WAYS, SACQ, PSS, and 4 COVID-19 adjustment domains. More details about these questionnaires are described in the Methodology section. Next, we developed separate machine learning models for the Asian-American (104 participants) and non-Asian-American (297 participants) groups to classify well-adjusted and poorly adjusted students for each of the four COVID-19 domains based on students’ individual characteristics as revealed by WAYS, SACQ, and PSS results. Lastly, we extracted the principal risk factors using the SHAP method [15] and compared the different coping patterns between the Asian and non-Asian groups. The findings can help university administrators institute customized student support systems. These machine learning models and the SHAP method are described in the Machine Learning Methods section. In the following sections, we review the theories related to ways of coping, college adjustment, and perceived stress.

### 1.1. Theory of Ways of Coping

The transactional model of stress and coping [16] describes how individuals approach and cope with perceived stress. It identifies coping as a cognitive and behavioral effort that an individual engages in to manage the demands of a stressful situation, both internally and externally. The stressors and the utilized coping strategies are heavily dependent on the individual’s beliefs, constraints, demands, and environments [16]. The coping styles and strategies used by the individual also affect the consequences (i.e., level of anxiety, psychological distress) of the stressful events [12].

Lazarus and Folkman [16] identified eight coping strategies, namely, confrontive coping, distancing, seeking social support, accepting responsibility, positive reappraisal, planful problem-solving, escape-avoidance, and self-controlling. As the labels imply, those who engage in confrontive coping tend to utilize aggressive and even risky efforts to alter the stressful situation, whereas those who practice distancing tend to move away from the stressors. Individuals who seek social support tend to reach out to others for consultation and comfort, and those who accept responsibility focus on reclaiming self-worth and engaging in positive changes. Positive reappraisal identifies individuals who try to focus on personal growth to create positive meanings from stressful events, and planful problem-solving is used by individuals who deliberately try to change the situation by focusing directly on the problem itself. Lastly, individuals who engage in escape-avoidance tend to utilize wishful thinking and behaviors to escape from the stressor, and those who engage in self-controlling behaviors tend to regulate their own feelings and actions in vivo [17]. Together, these eight coping strategies can be divided into three coping styles, which are emotion-focused, problem-focused, and avoidance-focused coping [16].

As one of the most widely acknowledged models of stress and coping [18], research has been conducted to examine its cross-cultural reliability and validity. Although limited, these studies have discovered that while Asian populations practice the coping strategies that Lazarus and Folkman [16] identified [19,20], Asian-American college students tend to report more avoidance coping strategies and less constructive thinking compared with their Caucasian-American counterparts [19] and are much less likely to seek social support compared with other racial/ethnic groups [19,21]. These results could be largely due to cultural differences and traditional values embedded in their Asian heritages [19,22,23].

### 1.2. Theory of SACQ

Transitioning to college is one of the most difficult changes a student will face in their lifetime [24]. The way in which a student meets the demands of college is called adjustment. Adjustment to college life is a process that students have to undergo when transitioning from high school to college. Not only is the transition process multifaceted in areas such as academic, social, and personal–emotional adjustment and university attachment, but the success of this transition affects the overall college experience and correlates with other life outcomes [13,24]. To better understand college students’ adjustment process and to identify at-risk students who need additional support, Baker and Siryk [13] developed the SACQ, which included aspects of academic adjustment, social adjustment, personal–emotional adjustment, and university attachment. By assessing aspects of students’ academic motivation, social environment, sense of psychological and physical well-being, and attachment to their institutions, as well as their overall levels of satisfaction in these four areas, the SACQ contributes to understanding college students’ adjustment levels as well as targeting their areas of need. Studies have shown that students who are able to develop overall positive experiences and relationships with their peers, faculty, and university and successfully adapt to college demands develop a better sense of social integration, navigate through university stressors more effectively, and achieve greater academic success [24,25].

Of note, the standardization samples of the SACQ included mainly Caucasian- and African-American university students across North America. It is unclear whether Asian students were also included in the samples [13]. Research that has attempted to investigate the reliability of the SACQ in measuring the effect of race on university students’ adjustment process found that SACQ scores showed a lack of difference between racial and ethnic groups [26,27], including those who identified as Asian, with the exception of the domain of personal–emotional adjustment [28].

### 1.3. Theory of Perceived Stress Scale

Stress emerges when an individual’s perceived demands in a situation are beyond their own capacity to deal with the circumstances [16]. Perceived stress is determined by an individual’s feelings about the general stressfulness of their life and their ability to work through such stress during a given period of time. In 1984, Lazarus and Folkman posited the transactional model of stress and coping, which defined stress as resulting from perceiving situational demands as beyond one’s resources and capabilities to address and cope with. According to Lazarus and Folkman [16], the level of stress elicited by the situation depends on the individual’s confidence and belief in how effectively they can navigate the situation in the context of their abilities and environments. Importantly, research has shown that different individuals may perceive the same stressful situations differently due to their levels of self-efficacy as well as their cognition styles [29,30]. Therefore, it is not uncommon that two individuals who encounter the same stress-provoking situations, such as the COVID-19 pandemic and anti-Asian discrimination, can have very different stress responses.

### 1.4. Research Questions and Originality of the Research

In order to examine how the way of coping, college adjustment, and perceived stress affected university students’ responses to the COVID-19 pandemic, we formed the following questions. The first research question focused on studying the differences between Asian and non-Asian groups in their responses within four domains after the onset of the pandemic: academic adjustment, emotional adjustment, social support, and discriminatory impacts related to COVID-19. The second research question was to examine whether students’ individual characteristics, as measured by the WAYS, the SACQ, and the PSS, could predict academic adjustment, emotional adjustment, social support, and discriminatory impacts related to COVID-19 among Asian and non-Asian students and to explore the differences between the two groups. This study utilized six established machine learning methods and compared their efficacy for our predictive tasks. The analysis approaches based on machine learning were novel in the context of examining psychological and emotional responses to COVID-19 among university students.

## 2. Materials and Methods

Institutional Review Board (IRB) approval was obtained from Fordham University in compliance with the American Psychological Association [31] guidelines for ethical research. This study is based on data collected from March to June 2020 during the initial peak of the COVID-19 pandemic. In the original data collection, an eligible participant was (a) at least 18 years old and (b) enrolled as an undergraduate or a graduate/professional student in a college or university within the United States.

### 2.1. Student Adaptation to College Questionnaire

The SACQ is a 67-item self-report measure that assesses 4 components of college adjustment, namely, academic adjustment (AA; α = 0.88), social adjustment (SA; α = 0.91), personal–emotional adjustment (PEA; α = 0.87), and university attachment (UA; α = 0.90). Academic adjustment examines students’ adaptation to their university’s educational demands, while social adjustment focuses on students’ adjustment to interpersonal and social demands related to their university experiences. Personal–emotional adjustment measures students’ psychological and physical well-being, and university attachment assesses students’ level of commitment, agreeableness, and connection to their university’s environments, beliefs, and goals [13,32]. Participants responded using a 9-point Likert scale (1 = Doesn’t apply to me at all; 9 = Applies very closely to me), and the participants’ responses for each subscale were totaled and converted into t-scores to obtain their levels of college adjustment.

### 2.2. Perceived Stress Scale

The PSS [14] is a 10-item self-report measure developed to evaluate an individual’s perceived degree of stress when encountering specific events. The questions were designed to be of a general nature to not only determine the individual’s overall current level of stress but also enable the scale’s usage across different populations and life scenarios. The respondents were asked to rate how often they felt or thought in a particular way within the last 30 days on a 5-point Likert scale (0 = Never; 4 = Very Often). The scores of the 10 questions were totaled to obtain a psychological stress score. The higher the psychological stress score, the greater the level of stress that the respondents perceived in the last 30 days.

### 2.3. Ways of Coping Questionnaire

The WAYS is a 66-item self-report questionnaire that measures participants’ most frequently practiced coping strategies when placed in stressful situations [12]. This widely used coping measure [18] was selected specifically to measure participants’ use of coping strategies during the early stages of the COVID-19 pandemic. With a 4-point Likert scale ranging from “does not apply or not used” to “used a great deal”, the questionnaire totaled the scores of the participants’ responses to generate 8 different subscales (i.e., confrontive coping, distancing, self-controlling, seeking social support, accepting responsibility, escape avoidance, planful problem-solving, positive reappraisal; [16]), with higher scores indicating higher usage of the coping behaviors in that subscale to cope with COVID-19-related stressors.

### 2.4. COVID-19 Questionnaire

The COVID-19 questionnaire was a self-report questionnaire developed to examine participants’ responses and adjustments to the COVID-19 pandemic. This 5-point Likert-scale self-report was an adaptation of an unpublished instrument designed to measure the experiences and mental health of Chinese university students during the initial COVID-19 outbreak in China [33]. Four subdomains were based on the factor analyses completed on the original questionnaire, namely, academic adjustment, emotional adjustment, social support, and discriminatory impacts related to COVID-19. The higher the scores on these subdomains, the better the adjustment of the participants during the COVID-19 pandemic.

### 2.5. Machine Learning Methods

To build our machine learning models, we first labeled each participant in each study group as well-adjusted (class 1) or poorly adjusted (class 0) in each of the four COVID-19 adjustment domains. To accomplish this, we identified the set of questions, *Q,* in the survey pertinent to each domain and computed the average score of answers to these questions. A participant was labeled as a class 1 instance if their total score for *Q* was above the average. Otherwise, the participant was labeled as a class 0 instance. Table 1 presents the distribution of participants for each of our classification tasks for the Asian and non-Asian groups.

The input features of our classification models are the students’ scores in each subscale for the WAYS, SACQ, and PSS domains. The total number of features is 13, with 8, 4, and 1 from WAYS, SACQ, and PSS, respectively. Table 2 presents the list of features employed by the predictive models and their brief descriptions.

We employed six established machine learning methods and compared their efficacy for our predictive tasks, namely, logistic regression (LR [34]), support vector machine (SVM [35]), decision tree (DT [36]), random forest (RF [37]), neural network (NN [38]), and AdaBoost [39]. We leveraged their implementations from Python’s scikit-learn package [40]) All models were trained using a 10-fold (outer) cross-validation. Therein, we divided the training data into five disjoint partitions (i.e., folds) and trained/evaluated each classifier five times with different training and test data. Specifically, at each iteration, *i* (*i* = 1, 2, … 10), fold *i* was designated as the test data, and the remaining nine folds were designated as the training data. We reported the average performance of the 10 test folds. Hyperparameters were selected using a nested 5-fold (inner) cross-validation on the training data.

### 2.6. Evaluation Metrics

We evaluated the performance of our classification models using six metrics:

- Overall accuracy: The fraction of correctly classified instances in the test data.
- Recall: The fraction of correctly classified instances among all well-adjusted instances.
- Specificity: The fraction of correctly classified instances among all not-well-adjusted instances.
- Precision: The fraction of correctly classified instances among all positive predictions.
- F1 score: Harmonic mean of recall and specificity.
- AUC score (of the ROC curve)*:* A ROC curve displays the trade-off between the true positive rate (TPR, or sensitivity) and the true negative rate (TNR, or specificity) of a classification model at different threshold settings. The AUC score reveals the capability of a model to separate positive and negative classes; that is, the higher the AUC score, the more effective a model is at performing the classification.

### 2.7. Model Interpretation with SHAP Plots

**Figure 1:**
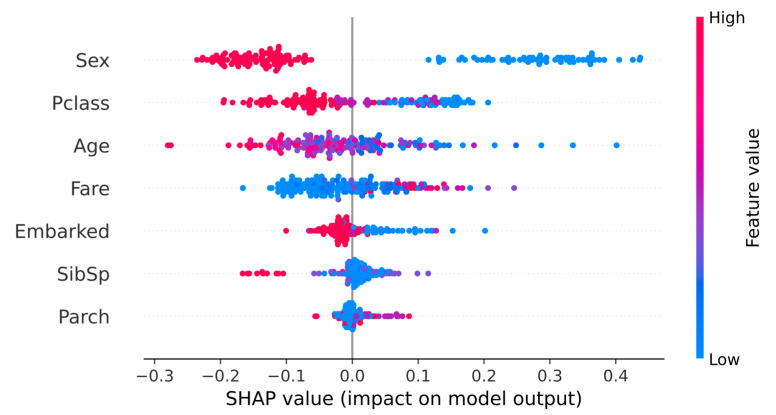
SHAP plot for titanic survival analysis using the random forest model.

SHAP (SHapley Additive exPlanation) is a framework to interpret the predictions of machine learning models [15]. In particular, a model is viewed as a coalition game in which accurate prediction is the goal, and the predictive features serve as the players. A SHAP plot provides an effective method to visualize the individual player’s contributions to the game’s outcomes. For example, Figure 1 illustrates a beeswarm SHAP plot for a random forest model applied to predicting a passenger’s survival status in the tragic Titanic accident. The dependent variables are 12 characteristic features (Sex, Pclass, Age, etc.) of each passenger. Figure 1 plots the predictive features in their order of relative importance along the y-axis. Each row illustrates a feature’s contribution to the predictive outcomes in which each dot represents an instance in the dataset. Feature values are color-coded from blue (low) to red (high). We observe that a passenger’s sex (encoded as male = 0 and female = 1) is the most predictive feature in predicting a passenger’s chances of survival. Low values (i.e., female) are concentrated on the right side of the y-axis, indicating a high probability of survival. The same is true for passengers with low age and low Pclass (encoded as first-, second-, and third-class cabins) values. Thus, we can infer that women, children, and passengers in first-class cabins had a greater chance of survival than the others.

In our study, we employed the SHAP approach to study the differences in relative importance and directional impact of those predictive features for the Asian and non-Asian groups in each classification task.

## 3. Results

This section presents the results of our classification tasks. The performance for each classification task was evaluated using the six evaluation metrics described above. The analysis focuses exclusively on the performance of the test data. For each study group and classification task, we present the results of the most effective model in terms of an AUC score. Risk factors and their directional contributions to the model output are extracted using the SHAP method for each model. A comparative analysis between the Asian and non-Asian groups is presented in the Analysis of Predictive Features section.

### 3.1. Classification Task Performance

#### 3.1.1. Academic Adjustment

Table 3 shows that random forest is the best model for predicting academic adjustment for both the Asian and non-Asian groups. Both groups achieved 85% overall accuracy and a 0.85 AUC score. For the well-adjusted and not-well-adjusted classes, the Asian group showed 91% (i.e., recall) and 79% (i.e., specificity) accuracy, respectively. In comparison, the non-Asian groups achieved 88% and 81%, respectively. Both groups showed similar precision and F1 scores.

#### 3.1.2. Emotional Adjustment

Table 3 shows that logistic regression is the most effective model in predicting student emotional adjustment for the Asian group and random forest is best for the non-Asian group. The results also suggest that the classification task is easier for the Asian group, with an 82% overall accuracy, a 0.82 AUC score, and 84% and 80% for recall and specificity, respectively. For the non-Asian group, the overall accuracy is 73%, with 71% and 76% for class 1 and class 0, respectively. The AUC score, precision, and F1 score are 0.73, 76%, and 0.73, respectively.

#### 3.1.3. Social Support

The results in Table 3 show that random forest is the best model for predicting student social support adjustment for both groups. For the Asian group, the model achieved a 72% overall accuracy with 70% and 75% for the well-adjusted and not-well-adjusted classes, respectively. The precision, F1, and AUC scores are 0.72, 0.69, and 0.72, respectively. For the non-Asian group, the overall accuracy is 72%, with 72% and 73% for class 1 and class 0, respectively. The precision, F1, and AUC scores are 0.74, 0.72, and 0.72, respectively.

#### 3.1.4. Discriminatory Impacts Related to COVID-19

Table 3 shows that random forest is most effective in predicting Asian students’ adjustment to discriminatory impacts during COVID-19. The overall accuracy for the classification task is 0.69, with 0.65 and 0.73 for class 1 and class 0, respectively. The AUC score, precision, and F1 score are 0.69, 0.7, and 0.67, respectively. In contrast, the SVM is most effective for the non-Asian group, and the results are similar to the Asian group.

### 3.2. Analysis of Predictive Features

Another essential goal of this study is to identify the most relevant risk factors associated with the Asian and non-Asian groups and compare their differences. To this end, we extracted the top 10 predictors using the SHAP algorithm based on the best-performing models (Table 3) and ranked them by their magnitude of contribution to the model outcome. Table 4 presents the predictive features for each study group and classification task. The bold type indicates variables whose SHAP values are above the average magnitude. Futhermore, corresponding SHAP plots are presented in each subsection to visualize the directional impact of these predictors on each target variable. We are particularly interested in distinguishing the positive and negative predictors and their differences between the Asian and non-Asian groups.

#### 3.2.1. Academic Adjustment

**Figure 2:**
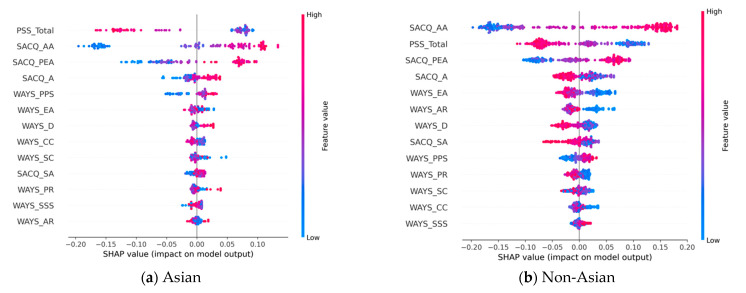
SHAP plots for academic adjustment based on the most effective models: (**a**) SHAP plot models of academic adjustment for Asian-American students; (**b**) SHAP plot models of academic adjustment for non-Asian-American students.

In predicting student academic adjustment during the COVID-19 pandemic, Table 4 shows that the features with above-average contributions (i.e., bold type) to the models’ predictions are the same for the Asian and non-Asian groups. However, the PSS_total is more important for the Asian group when compared with SACQ_AA for the non-Asian group. We further studied the directional impact of these top features using the corresponding SHAP plots of the two models for the Asian (Figure 2a) and non-Asian (Figure 2b) groups. A closer examination reveals that the PSS_total is a negative predictor, meaning that a higher stress level (red) is more likely to be associated with a negative adjustment to academic changes (i.e., the left region of the y-axis). In contrast, SACQ_AA and SACQ_PEA are positive predictors; that is, their values are associated with positive academic adjustment outcomes.

Among the remaining variables, SACQ_A and WAYS_D showed opposite directional contributions to the two study groups. Specifically, SACQ_A is a positive predictor for the Asian group, but its contribution to the non-Asian group is negative. The same observation can be made for WAYS_D.

#### 3.2.2. Emotional Adjustment

**Figure 3:**
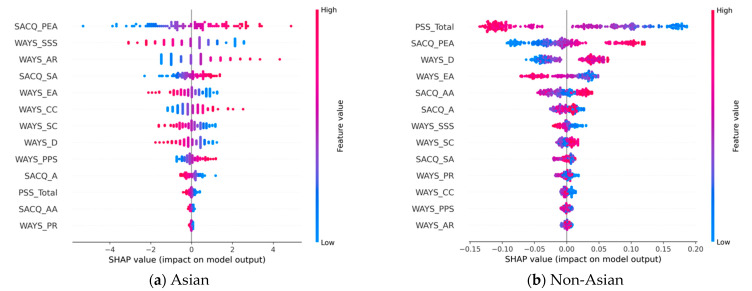
SHAP plots for emotional adjustment based on the most effective models: (**a**) SHAP plot models of emotional adjustment for Asian-American students; (**b**) SHAP plot models of emotional adjustment for non-Asian-American students.

In predicting student emotional adjustment during the pandemic, Table 4 shows that there are six and four above-average contribution (i.e., bold) features for the Asian and non-Asian groups, respectively. Of these, SACQ_PEA and WAYS_EA are common to the two groups. An analysis of the two SHAP plots in Figure 3 suggests that the directional impact of SACQ_PEA is positive while WAYS_EA is negative for both study groups. In addition, the PSS_total is the most important predictor for the non-Asian group, while its contribution to the Asian group is limited. Nevertheless, the PSS_total demonstrates the same directional impact for both groups; that is, a higher stress level is likely to be associated with a class 0 (i.e., not-well-adjusted) prediction. Lastly, WAYS_AR, SACQ_SA, and WAYS_CC are significant positive predictors for the Asian group. However, their contributions are limited for the non-Asian group.

#### 3.2.3. Social Support

**Figure 4:**
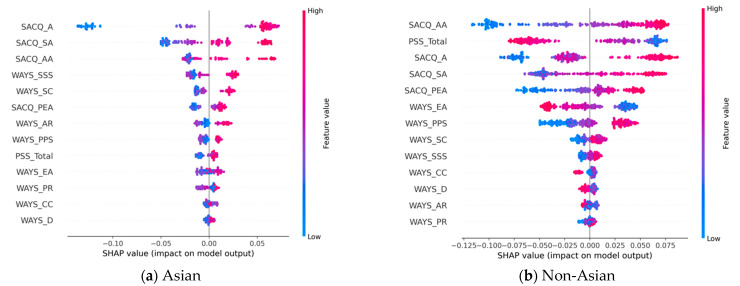
SHAP plots for social support based on the most effective models: (**a**) SHAP plot models of social support for Asian-American students; (**b**) SHAP plot models of social support for non-Asian-American students.

Table 4 shows that there are four and seven significant predictors for the Asian and non-Asian groups, respectively. Of these, SACQ_A, SACQ_SA, and SACQ_AA are the overlapping principal predictors for both groups. The SHAP plots in Figure 4 show that they are all positive contributors to the two models’ outputs. In addition, the PSS_Total is a unique principal predictor for the non-Asian group. The directional contribution of the PSS_Total is negative for the non-Asian group while positive for the Asian group. A similar pattern is found for WAYS_AR and WAYS_D, although their contributions to the models’ decisions are limited. Lastly, WAYS_EA is a negative predictor for the non-Asian group while its contribution to the Asian group is limited. SACQ_PEA and WAYS_PPS exhibit the same positive contribution to both groups, although they are principal predictors only for the non-Asian group.

#### 3.2.4. Discriminatory Impacts Related to COVID-19

**Figure 5:**
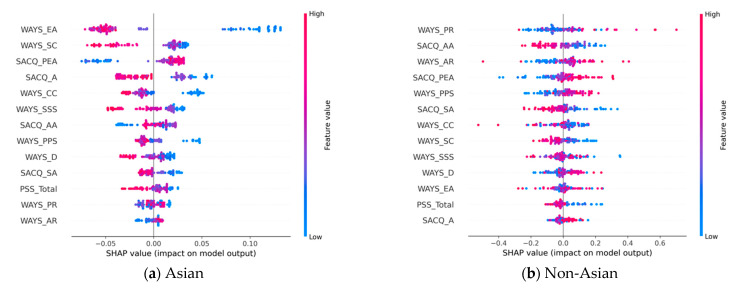
SHAP plots for discriminatory impact based on the most effective models: (**a**) SHAP plot models of discriminatory impact for Asian-American students; (**b**) SHAP plot models of discriminatory impact for non-Asian-American students.

In predicting student adjustments to discriminatory events during the pandemic, Table 4 shows that there are six and seven above-average contribution (i.e., bold) features for the Asian and non-Asian groups, respectively. Of these, SACQ_PEA and WAYS_CC are common to the two groups. An analysis of the corresponding SHAP plots in Figure 5 reveals the following key findings:

- WAYS_EA and WAYS_SC are the top predictors for the Asian group, and they are negatively correlated with the class 1 (i.e., well-adjusted) outcomes. However, both features are insignificant for the non-Asian group.
- WAYS_PR and SACQ_AA are the top two predictors for the non-Asian group and display a positive and negative correlation to class 1 (i.e., well-adjusted), respectively. However, both features are insignificant for the Asian group.
- For the two common significant features, SACQ_PEA is a positive predictor, and WAYS_CC is a negative predictor for both groups.
- SACQ_A exhibits an opposite directional impact on the two study groups. In particular, it is a significant, negative risk factor for the Asian group. However, it is positively correlated to class 1 for the non-Asian group, but it is an insignificant feature for this group. A similar pattern is found for WAYS_SSS, whose impact on the Asian group is negative while its overall trend for the non-Asian group is positive.
- WAYS_PPS also exhibits an opposite directional impact on the two study groups. It is a significant positive risk factor for the non-Asian group. However, it is negatively correlated to class 1 for the Asian group, but it is an insignificant feature for this group.

## 4. Discussion

### 4.1. Academic Adjustment

Perceived stress, or how participants feel about their own ability to handle stressors during COVID-19, dictated participants’ stress levels in this study [14]. Our findings suggest that perceived stress regarding academic adjustment is more important for the Asian group than it is for the non-Asian group. As a group, Asian Americans have been found to perform better academically than other subpopulations but also have higher levels of fear of academic failure and spend more time studying than their non-Asian peers [23,40,41], suggesting the importance of perceived stress for these students. Our findings indicate that perceived stress, college academic adjustment, and college personal–emotional adjustment are also positive predictors of COVID-19 academic adjustment for both Asian and non-Asian students, stressing the importance of academic and personal–emotional stress factors. College attachment and distancing coping are negative predictors of COVID-19 academic adjustment in both groups. This finding suggests that high attachment to one’s institution might not serve as a protective factor in terms of COVID-19 challenges. In addition, distancing coping (i.e., the individual removing themself from the situation) is not beneficial for COVID-19 academic adjustment.

### 4.2. Emotional Adjustment

University personal–emotional adjustment is a positive predictor for both groups. The stress of navigating the unknown and uncontrollable situations associated with the initial peak of the COVID-19 pandemic might be related to previous adjustments to personal–emotional demands and stressors associated with university experiences. Experiences related to personal–emotional adjustment with regard to the university experience may have been beneficial in managing perceived stress related to the COVID-19 pandemic. The coping literature defines avoidance-focused coping as a typical initial reaction to stress [42]. However, such avoidance-focused coping strategies emerged as a negative predictor for both groups of students in this study. The findings suggest that the coping strategies of accepting responsibility and confrontive coping are negative predictors for the Asian group. It is possible that the non-Asian sample was newly dealing with the COVID-19 pandemic during the initial outbreak of the virus in the United States, while the Asian sample had become more accustomed to news of the virus due to connections or tracking pandemic-related news overseas. Adopting a coping strategy of accepting responsibility to confront the COVID-19 pandemic, which is essentially uncontrollable at the individual level, might be counterproductive for Asian students.

### 4.3. Social Support

The results also show that academic and social adjustment to college, as well as students’ level of attachment to their universities, are overlapping positive predictors of how both Asian and non-Asian college students adjusted socially during the COVID-19 pandemic. This means that the better adjusted both Asian and non-Asian students are academically and socially in their university environments, or the better attached these students are to their universities, the better socially adjusted they are during the COVID-19 pandemic. This finding is corroborated by the existing literature, which found that students who reported better university attachment and satisfaction tended to experience better social support and academic competence [43], and securing social support in turn indicated lower levels of stress experienced during the adjustment processes [44].

However, different results emerged when comparing the COVID-19 social support adjustment between the Asian and non-Asian student groups. For instance, for the non-Asian student group, the higher the stress level perceived, the more engagement in escape avoidance and planful-problem-solving coping strategies. Furthermore, better adjustment to personal–emotional demands in college led to a lower level of adjustment socially during the COVID-19 pandemic. For the Asian student group, conversely, perceived stress is a positive predictor of how well they adjusted socially to the pandemic. Likewise, personal–emotional adjustment to college and coping strategies such as escape-avoidance and planful problem-solving have a very limited effect on determining the Asian student group’s level of social adjustment during the pandemic. This difference between student groups could be explained by their prior experiences with racially discriminatory incidents. As discussed in past research, social support is only effective in coping with racially discriminatory incidents when the person presents with a moderate-to-high external locus of control, which may reflect the person’s cultural perceptions and real-life experiences. The locus of control, specifically, is defined as how much the person believes they have control over the situation as opposed to being controlled by external factors [45]. For Asian students, it is highly likely that they had experienced racial discriminatory incidents that were not well acknowledged in the past [46,47], and this might not be applicable to non-Asian students. This could create heightened levels of perceived stress as well as higher usage of various coping strategies by Asian students and increase the likelihood of them seeking social conformity and support. Therefore, when witnessing or experiencing COVID-19-fueled anti-Asian racism, it is possible that non-Asian students are less able to adjust socioemotionally to these incidents and are uncertain about how to approach these incidents, whereas Asian students have already had prior experiences doing so.

### 4.4. Discriminatory Impacts Related to the COVID-19 Pandemic

Several interesting results emerged from the analyses regarding Asian and non-Asian college students’ perceptions and reactions regarding COVID-19-fueled discriminatory incidents. First, Asian and non-Asian student groups utilized different coping strategies when encountering such incidents. Specifically, escape-avoidance coping and self-controlling coping strategies are the top two negative predictors for the Asian student group’s reactions, yet these were insignificant for the non-Asian student group. This means that the more the Asian student group engaged in escape-avoidance and self-controlling strategies, the less impacted they were by the discriminatory incidents. This is likely due to the specific nature of COVID-19-fueled anti-Asian racism. Although Asian Americans have likely experienced other forms of racism in the past [47,48,49], COVID-19-fueled anti-Asian racism has been so unique that the Asian-American community may not have been prepared for it. Not knowing how to approach and process these racist incidents might lead to higher engagement in avoidance-coping strategies or coping strategies that focus on alleviating personal unpleasant emotions [48]. As indicated by past research, engaging in avoidance-coping strategies when encountering discriminatory incidents could be problematic in the long term for stigmatized minority groups such as Asian Americans, since it may heighten their perception of help-seeking stigma, which then further increases their engagement in avoidance-coping strategies [50,51].

On the contrary, the non-Asian student group reported feeling more impacted by the discriminatory incidents if they engaged in more positive-reappraisal coping and planful-problem-solving coping strategies, and/or were less well-adjusted to their colleges’ academic demands, which are all insignificant indicators for the Asian student group. According to past research, this is likely due to several reasons. Asian Americans are aware of society’s positive perceptions as well as the discriminatory treatment they receive despite the common “model minority” belief that society has placed upon them. Therefore, expecting Asian Americans to foster positive meaning and self-growth or plan to resolve the problem when encountering a stress-inducing situation, such as COVID-19-fueled hate crimes, may not be as applicable and appropriate when compared with their non-Asian-American counterparts due to the heightened awareness of this paradox [52]. Additionally, Asian Americans have been found to perform better academically compared with other subpopulations while also having a higher level of fear of academic failure [23]. This could have contributed to the insignificant impact of college academic adjustment on Asian-American students’ perceptions and feelings regarding COVID-19-fueled anti-Asian racism, as college academic demands may be considered a separate factor by these students when processing their thoughts and feelings.

For both Asian and non-Asian student groups, our findings suggest that students reported better adjustment to feelings and experiences elicited by COVID-19-fueled hate crimes if they had better personal–emotional adjustment during college transitions or if they engaged in fewer confrontive coping strategies. Moreover, when the two groups were compared, it was found that Asian students reported more difficulties adjusting to COVID-19-related discriminatory incidents if they were more positively attached to their colleges or more prone to seek out social support. Both trends, although insignificant, positively predicted such experiences for non-Asian students. This could be due to the unexpected intensity of COVID-19-fueled anti-Asian racism, causing pre-existing protective factors such as college attachment and social support, as well as different coping mechanisms, to become ineffective. The differences between the coping strategies utilized by Asian and non-Asian student groups were also supported by the past literature regarding cultural differences in stress and coping [53,54,55,56]. As highlighted by Lu and Wang [45], coping strategies are most effective when matched with the individual’s belief system. Regardless of the intensity of anti-Asian racism or the students’ adjustment process, it is important to keep in mind that cultural differences and help-seeking stigma can lead to students’ selecting different coping strategies [50,51]. In addition, college adjustment and social support play a crucial protective role in ethnic minority students’ experiences in college, as they may experience less discrimination, social devaluation, and adjustment difficulties when more social support from faculty, peers, and institutions is in place during transitions [52].

### 4.5. Limitations and Future Work

Due to the nuance of COVID-19 and its unique impact, specifically on university students and the Asian-American population, a measure was created to examine COVID-19-related factors for the original data collection. This COVID-19-related questionnaire may not have comprehensively investigated the four domains of the measure (i.e., academic adjustment, emotional adjustment, social support, and discriminatory impacts related to COVID-19).

The SACQ instrument measured the multifaceted college adjustment constructs. Although the measure has been proven to demonstrate consistency and validity [13], the lack of attention to diverse populations in its creation, as well as consideration of the relationship between factors such as culture in college adjustment, prevents the generalization of findings. The SACQ measure was originally validated primarily with White students in the United States and has not been studied empirically with other populations, such as Asian samples. Future research should consider determining the suitability and appropriateness of SACQ for diverse populations as well as for graduate students.

In our approach, we studied the four psychological adjustment domains independently using standard, well-established machine learning models. However, a student’s behavior in these four domains could be interconnected. For instance, a student’s emotional state could affect their academic adjustment. Thus, a valuable future undertaking could be to exploit the interrelationship among the different predictive tasks. More advanced machine learning techniques, such as multitask learning [57], could offer a promising solution.

## 5. Conclusions

The current study explored and examined the experiences and ways of coping, perceived stress, and adjustment of university students during the COVID-19 pandemic, specifically those of Asian-American undergraduate and graduate students through a machine learning approach.

In short, our findings suggested that perceived stress regarding academic adjustment is more important for the Asian group than it is for the non-Asian group. University personal–emotional adjustment is a positive predictor for both groups. Asian and non-Asian student groups applied different coping strategies when encountering COVID-19-related discriminatory incidents. For both groups, students reported better adjustment to feelings and experiences elicited by COVID-19-fueled hate crimes if they had better personal–emotional adjustment or if they engaged in fewer confrontive coping strategies. When uncontrollable public health emergencies occur, university mental health service providers might consider strategies to reduce perceived stress among university students, provide services that could promote better personal–emotional adjustment, encourage alternative approaches to replace confrontive coping strategies, and need to be mindful of student differences that could be related to their racial and cultural backgrounds.

Our findings provide insights into the risk factors and their directional impacts affecting Asian and non-Asian students’ well-being during the pandemic. Future research should continue to explore the unique experience of Asian-American university students and their identity and growth in order to contribute to higher education research on this population as well as the Asian-American population in general. Although this study was conducted in the United States, the findings could bring insights into understanding university students’ experiences during a public health crisis (i.e., COVID-19) in other countries because university students share some similarities in terms of their academic demands and experiences in higher education. Psychological distress and coping difficulties associated with the COVID-19 pandemic have been documented in college students in other countries [58]; thus, experiences in the United States could be applicable to other cultures. University-level mental health service providers are suggested to provide a wide range of services, such as group-based mental health workshops that focus on prevention and psycho-education, focus-group-type counseling that centers on targeted groups of students, and individual-level counseling and services that stress individualized support. By using a systematic and programmatic approach to mental health service delivery, it is hoped that a wide range of university students will have access to such support, especially during an unprecedented public health crisis such as COVID-19.

## Figures and Tables

**Table 1:** Data distribution for Asian and non-Asian groups in each study domain.

COVID-19 Adjustment Domain	#Well-Adjusted (Class 1)	#Not Well-Adjusted (Class 0)
	Asian Group	
Academic	57	47
Emotional	50	54
Social Support	69	35
Discriminatory Impact	46	58
	Non-Asian Group	
Academic	174	123
Emotional	184	113
Social Support	180	117
Discriminatory Impact	120	177

**Table 2:** Input features for the classification models.

Index	Feature	Description
1	SACQ_A	Student’s level of affection and connection with their university
2	SACQ_AA	Student’s adaptation to the educational demands of their university
3	SACQ_PEA	Student’s psychological and physical well-being in their university experiences
4	SACQ_SA	Student’s interpersonal and societal demands in their university experiences
5	PSS_Total	Student’s perceived level of stress in the past 30 days
6	WAYS_AR	A coping strategy that uses efforts to reclaim self-worth by engaging in positive cognitive and behavioral changes
7	WAYS_CC	A coping strategy that uses efforts that are considered aggressive and risky
8	WAYS_D	A coping strategy that involves actions removing oneself from a stressful situation
9	WAYS_EA	A coping strategy that involves wishful thinking and pessimistic behaviors to avoid the stressful situation
10	WAYS_PPS	A coping strategy that utilizes conscious effort that targets the problem directly to change the stressful situation
11	WAYS_PR	A coping strategy that involves efforts in focusing on self-growth and creating positive meanings from the stressful situation
12	WAYS_SC	A coping strategy that involves efforts in regulating one’s own feelings and actions
13	WAYS_SSS	A coping strategy that involves attempts to reach out to social networks for consultation and validation

**Table 3:** Performance of the best machine learning model for each study group.

Group	Model	Accuracy	Precision	Recall	Specificity	AUC	F1
Academic Adjustment							
Asian	RF	0.85	0.84	0.91	0.79	0.85	0.87
Non-Asian	RF	0.85	0.83	0.88	0.81	0.85	0.85
Emotional Adjustment							
Asian	LR	0.82	0.82	0.84	0.80	0.82	0.82
Non-Asian	RF	0.73	0.76	0.71	0.76	0.73	0.73
Social Support							
Asian	RF	0.72	0.72	0.70	0.75	0.72	0.69
Non-Asian	RF	0.72	0.74	0.72	0.73	0.72	0.72
Discriminatory							
Asian	RF	0.69	0.70	0.65	0.73	0.69	0.67
Non-Asian	SVM	0.70	0.70	0.71	0.70	0.70	0.70

**Table 4:** Top 10 predictors for each classification task based on the most effective model.

Academic Adjustment		Emotional Adjustment		Social Support		Discriminatory Impact	
Asian	Non-Asian	Asian	Non-Asian	Asian	Non-Asian	Asian	Non-Asian
PSS_Total *	**SACQ_AA**	**SACQ_PEA**	**PSS_Total**	**SACQ_A**	**SACQ_AA**	**WAYS_EA**	**WAYS_PR**
**SACQ_AA**	**PSS_Total**	**WAYS_SSS**	**SACQ_PEA**	**SACQ_SA**	**PSS_Total**	**WAYS_SC**	**SACQ_AA**
**SACQ_PEA**	**SACQ_PEA**	**WAYS_AR**	**WAYS_D**	**SACQ_AA**	**SACQ_A**	**SACQ_PEA**	**WAYS_AR**
SACQ_A	SACQ_A	**SACQ_SA**	**WAYS_EA**	**WAYS_SSS**	**SACQ_SA**	**SACQ_A**	**SACQ_PEA**
WAYS_PPS	WAYS_EA	**WAYS_EA**	SACQ_AA	WAYS_SC	**SACQ_PEA**	**WAYS_CC**	**WAYS_PPS**
WAYS_EA	WAYS_AR	**WAYS_CC**	SACQ_A	SACQ_PEA	**WAYS_EA**	**WAYS_SSS**	**SACQ_SA**
WAYS_D	WAYS_D	WAYS_SC	WAYS_SSS	WAYS_AR	**WAYS_PPS**	SACQ_AA	**WAYS_CC**
WAYS_CC	SACQ_SA	WAYS_D	WAYS_SC	WAYS_PPS	WAYS_SC	WAYS_PPS	WAYS_SC
WAYS_SC	WAYS_PPS	WAYS_PPS	SACQ_SA	PSS_Total	WAYS_SSS	WAYS_D	WAYS_SSS
SACQ_SA	WAYS_PR	SACQ_A	WAYS_PR	WAYS_EA	WAYS_CC	SACQ_SA	WAYS_D

* Bold entries indicate features with above-average SHAP contribution.

## Data Availability

Due to privacy concerns mentioned in the IRB protocol, the data associated with this study cannot be provided to the public without the supervision of the researchers. However, individual researchers who are interested in obtaining access to the data for individual use are encouraged to contact the corresponding authors.
